# Supplementary material for: Cold deep subduction recorded by remnants of a Paleoproterozoic carbonated slab
Source: Nat Commun. 2018 Jul 17;9:2790. doi: 10.1038/s41467-018-05140-5 (PMC6050299; doi:10.1038/s41467-018-05140-5)
Supplement: Supplementary file 1 — Supplementary Information [file 41467_2018_5140_MOESM1_ESM.pdf]

# Cold deep subduction recorded by remnants of a Paleoproterozoic carbonated slab

Xu *et al.*

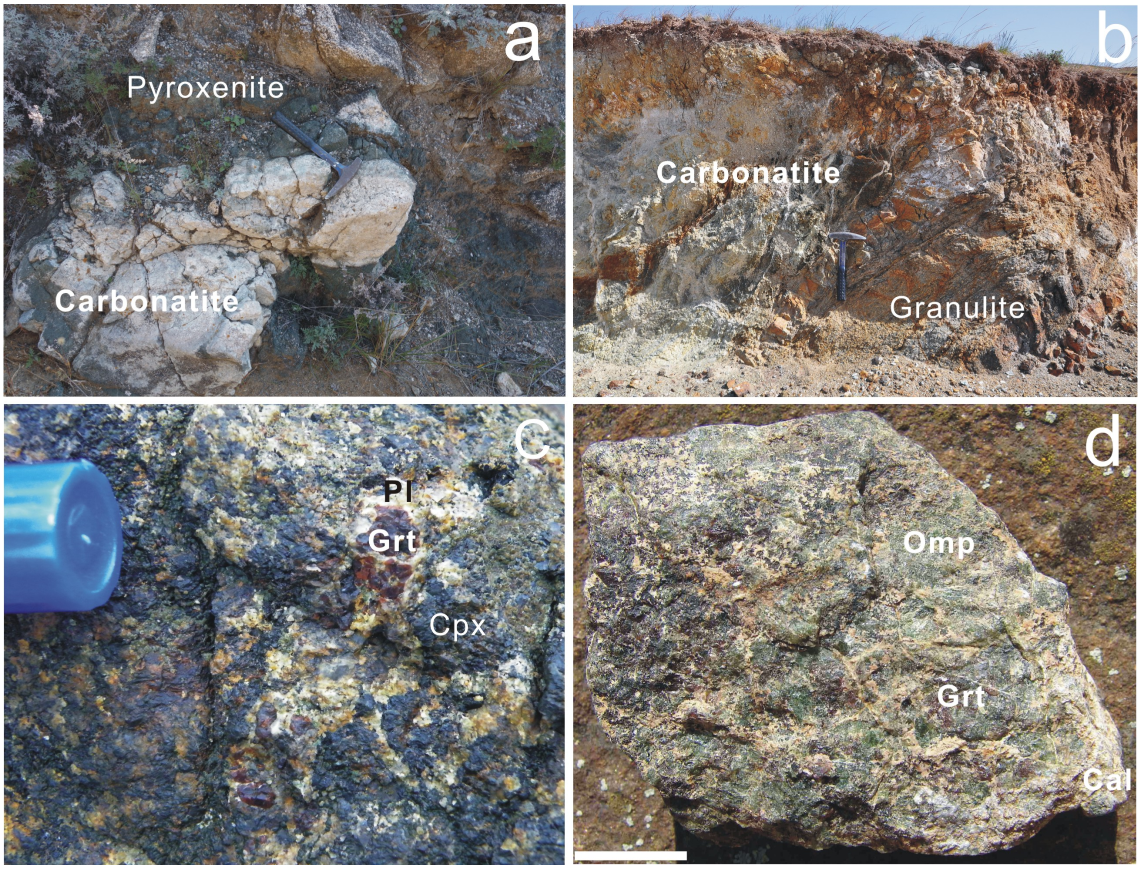


**Supplementary Figure 1: Photographs of carbonatite, granulite, and eclogite xenolith**. (a) Carbonatites are associated with pyroxenites. (b) Carbonatites are associated with granulites. (c) Granulite shows augen structure with plagioclase (Pl) and orthopyroxene (Cpx) around porphyroblastic garnet (Grt). (d) Eclogite hand-specimen is composed of garnet and omphacite (Omp), and contaminated by calcite (Cal). The scale bar is 10 mm.

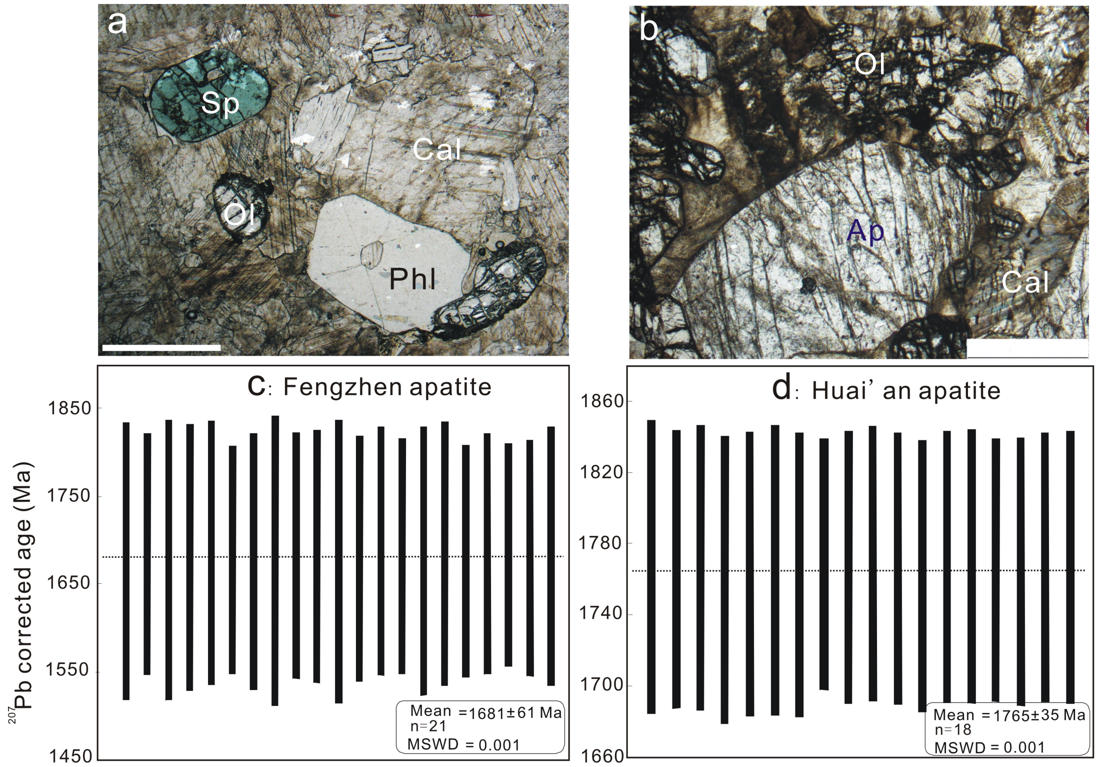


**Supplementary Figure 2: Characteristic minerals in the carbonatites and the ages determined from apatite dating**. (a) Representative photomicrograph of carbonatites shows calcite (Cal), primary olivine (Ol), phlogopite (Phl), and spinel (Sp). (b) Image shows apatite (Ap) in carbonatites used for dating. (c) Weighted U-Pb ages of $^{207}$Pb corrections of apatites are plotted for the Fengzhen area, with a mean age of 1681 Ma. (d) Weighted U-Pb ages of $^{207}$Pb corrections of apatites are shown for the Huai'an area, with a mean age of 1765 Ma. The scale bars in a and b are 500 μm.

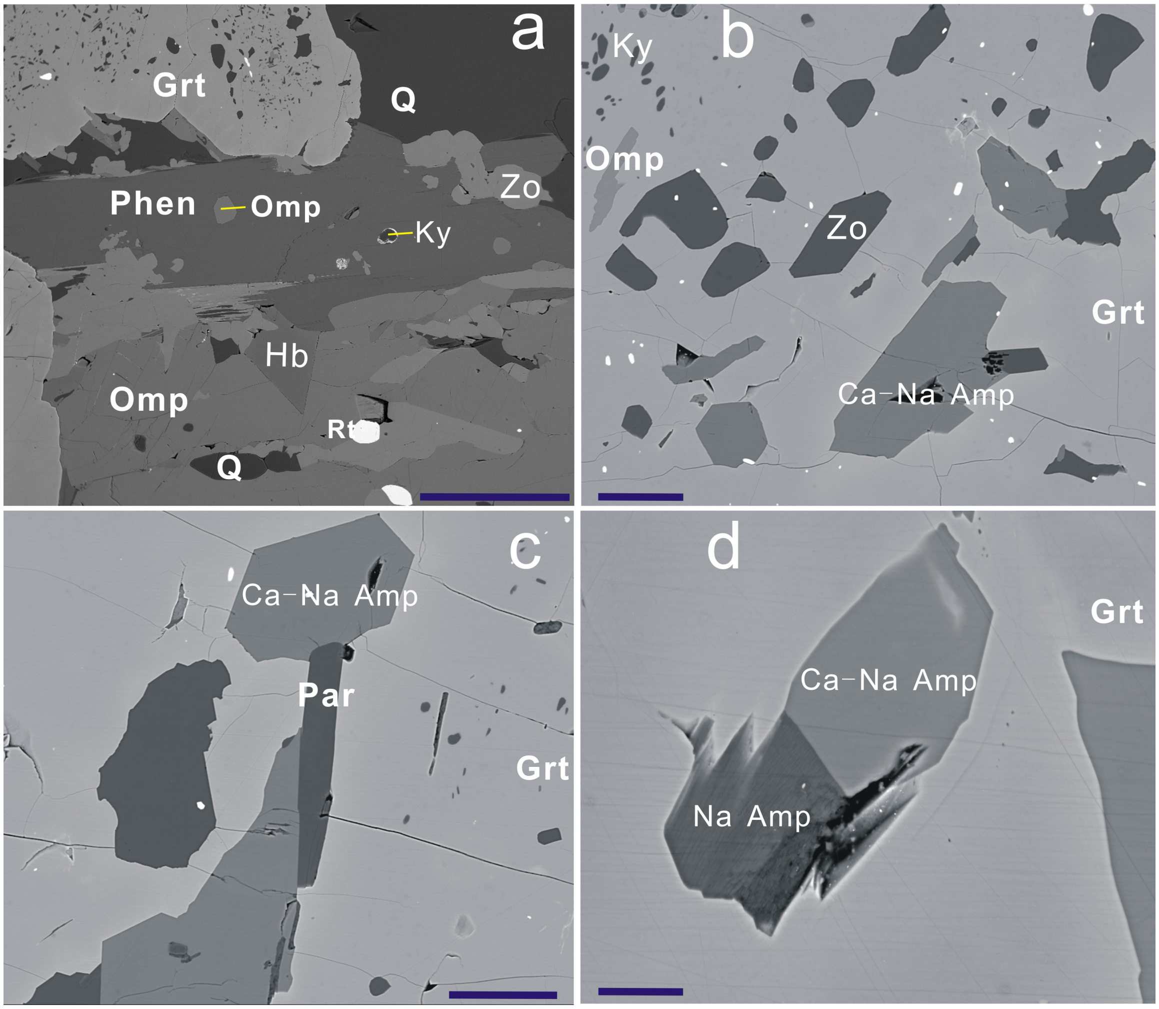


**Supplementary Figure 3: Representative back scattered-electron images showing characteristic textures of minerals in the eclogite xenolith**. (a) Representative matrix minerals, including omphacite (Omp), phengite (Phen), garnet (Grt), quartz (Q), and rutile (Rt). Phengite contains omphacite and kyanite (Ky) inclusions. Garnet hosts abundant mineral inclusions. (b) Selected inclusions in the garnet core show kyanite, omphacite, zoisite (Zo), and Ca-Na amphibole (Amp). (c) Garnet also contains minor Na-rich minerals such as paragonite (Par) along with Ca-Na amphibole. (d) Na-amphibole with Ca-Na amphibole is also observed as inclusion. The scale bars in a, b, c, and d are 500, 50, 50, and 10 μm, respectively.

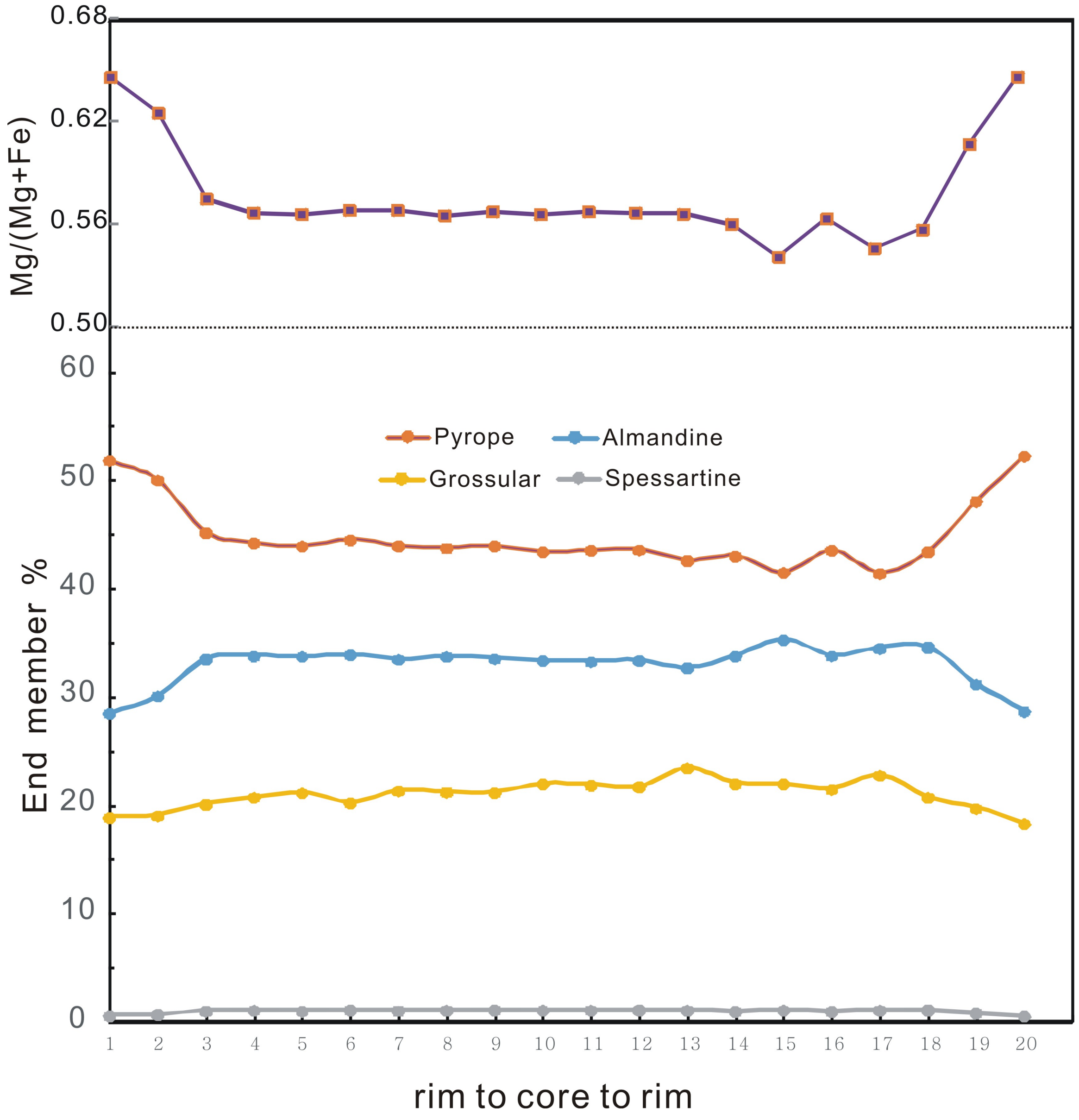


**Supplementary Figure 4: Rim-to-rim garnet compositional profile**. The profiles record increasing of pyrope and decreasing of almandine at the garnet rim, indicating a prograde metamorphism.

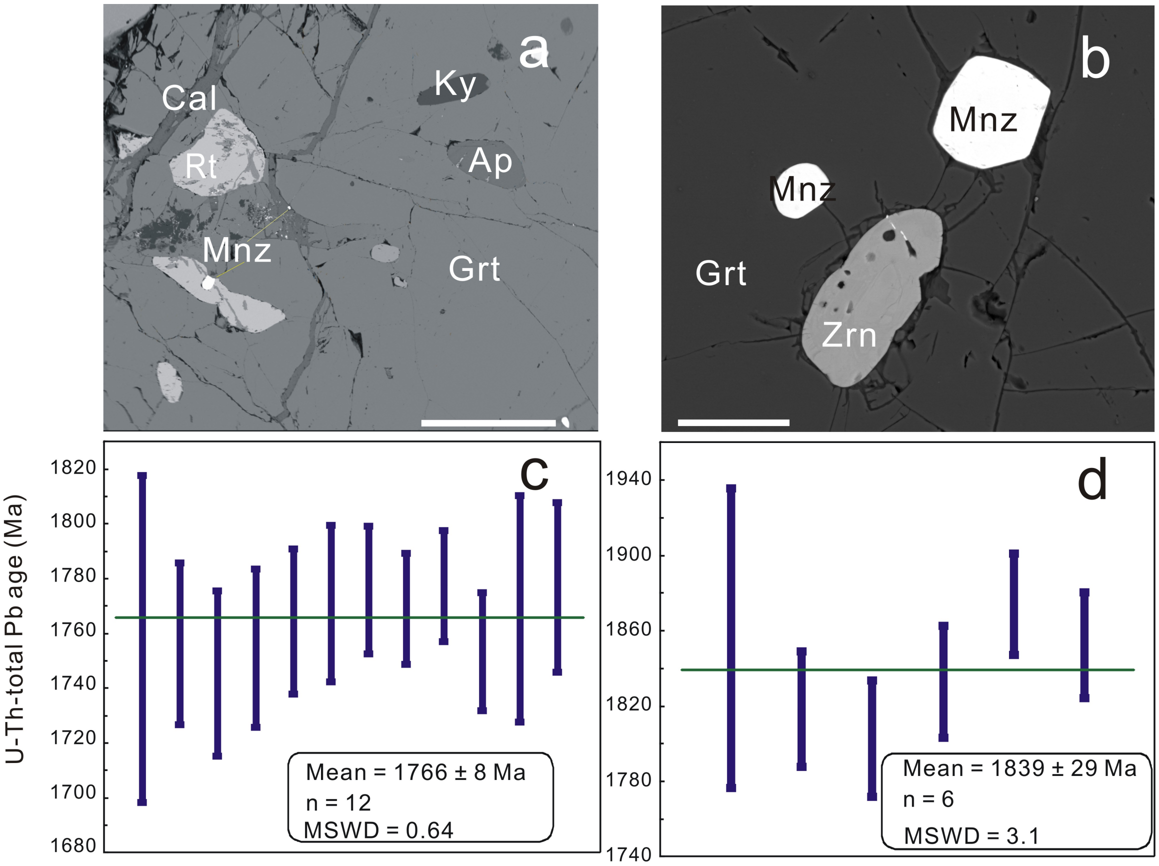


**Supplementary Figure 5: Monazite geochronology of eclogite xenolith**. (a) Monazites occur with calcite veinlets. (b) Monazites occur as primary inclusions in garnet. (c) Weighted U-Th total Pb ages of monazites from veinlets with a mean age of 1766 Ma. (d) Weighted U-Th total Pb ages of monazites from primary crystals with a mean age of 1839 Ma. The scale bars in a and b are 500 and 50 μm, respectively. Cal, calcite; Mnz, monazite; Rt, rutile; ky, kyanite; Ap, apatite; Grt, garnet; Zrn, zircon.

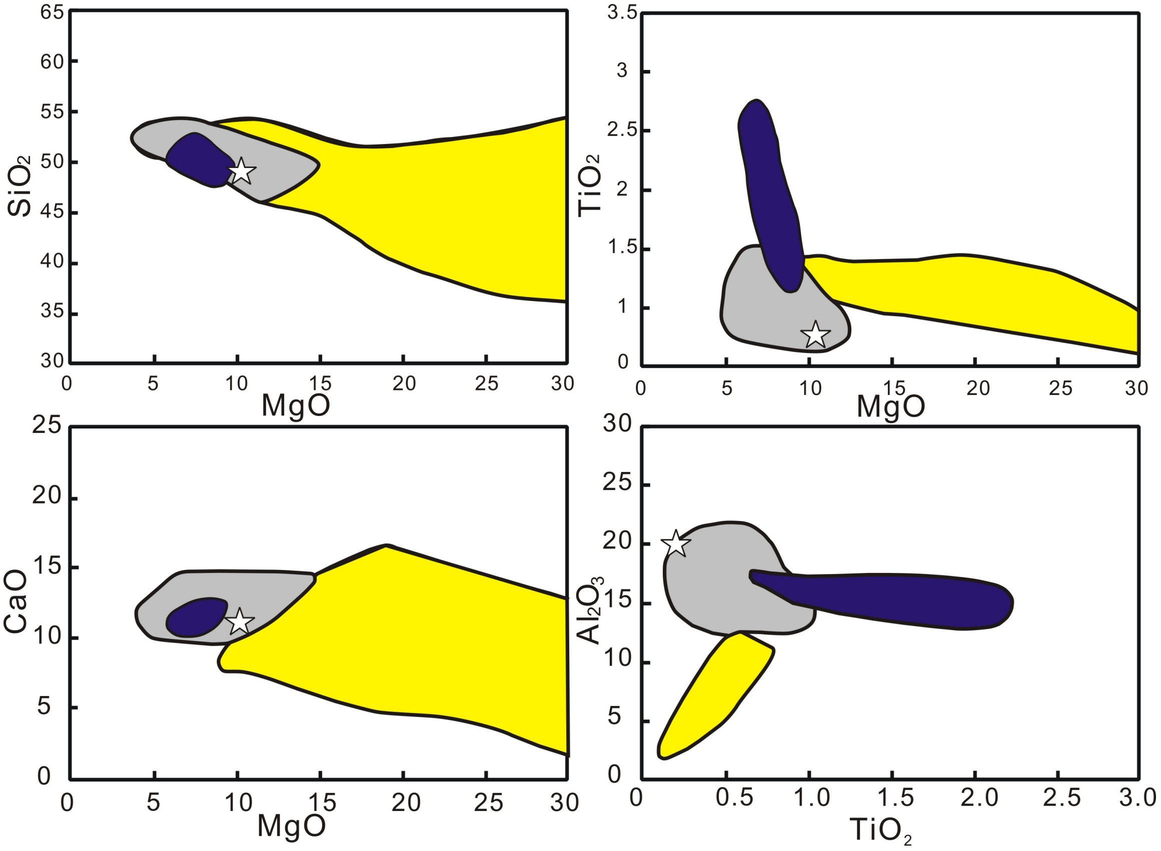


**Supplementary Figure 6: Major-element composition (wt.%) comparison of different type rocks.** The eclogite (star) has different $SiO_2$, $TiO_2$, CaO, MgO, and $Al_2O_3$ contents compared to those of komatiites (yellow) and MORB (blue), but it plots in the field of oceanic gabbros (grey)[1].

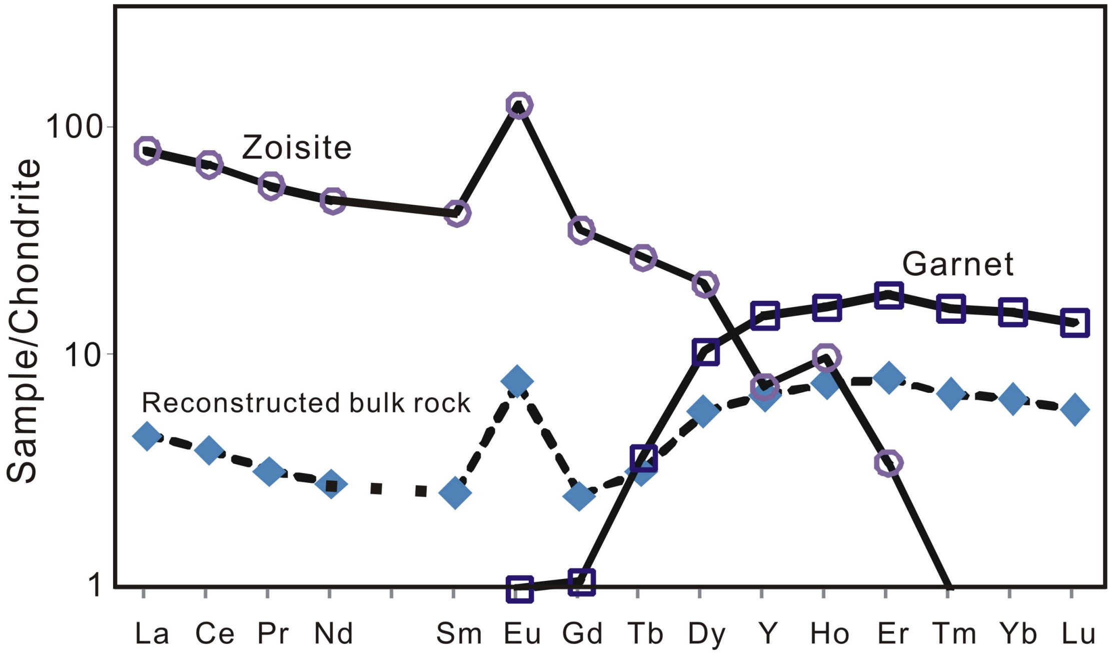


**Supplementary Figure 7: Chondrite-normalized REE pattern of zoisite, garnet and eclogite xenolith**. The REE content of reconstructed bulk rock is calculated by mineral compositions and modal mass shown in Supplementary Tables 5 and 6.

Supplementary Table 1: Compositions of minerals in carbonatites (wt%).

| Mineral | calcite(5)[a] | apatite(6) | phlogopite(5) | clinopyroxene(6) | olivine(6) | spinel(6) |
|---|---|---|---|---|---|---|
| $SiO_2$ | | 0.56(87)[b] | 39.99(121) | 52.50(174) | 41.86(41) | 0.02(1) |
| $TiO_2$ | | | 0.73(12) | 0.95(66) | 0.01(1) | bdl[c] |
| $Al_2O_3$ | | | 14.97(38) | 4.51(226) | 0.01(1) | 68.66(133) |
| FeO*[d] | 0.04(5) | 0.42(620 | 3.12(127) | 1.11(51) | 5.42(48) | 6.26(137) |
| MnO | 0.04(4) | 0.51(72) | 0.01(2) | 0.05(3) | 0.22(8) | 0.10(2) |
| MgO | 0.43(95) | | 24.55(116) | 16.59(68) | 52.59(40) | 24.78(95) |
| CaO | 54.07(117) | 51.82(123) | 0.02(4) | 25.88(39) | 0.04(3) | |
| BaO | 0.02(2) | | 1.24(59) | | | |
| SrO | 1.61(108) | 1.15(62) | | | | |
| $Na_2O$ | | 0.19(17) | 0.32(17) | 0.21(8) | | |
| $K_2O$ | | | 9.97(10) | bdl | | |
| $P_2O_5$ | | 41.01(82) | | | | |
| $La_2O_3$ | | 0.26(26) | | | | |
| $Ce_2O_3$ | | 0.78(101) | | | | |
| $CO_2$[e] | 43.63(29) | | | | | |
| F | | 4.10(49) | 3.68(41) | | | |
| $H_2O$ | | | 2.43(26) | | | |
| O=F | | -1.72(20) | -1.57(17) | | | |
| Total | 99.82(22) | 99.19(68) | 99.57(85) | 101.82(39) | 100.16(38) | 99.85(98) |
| *Cations*[f] | *3O* | *12(O,OH,F)* | *12(O,OH,F)* | *6O* | *4O* | *4O* |
| Si | | 0.049(77) | 2.857(32) | 1.869(66) | 1.004(11) | 0 |
| Ti | | | 0.040(7) | 0.025(18) | 0 | 0 |
| Al | | | 1.261(34) | 0.189(95) | 0 | 1.970(11) |
| $Fe^{3+}$ | | | | 0.037(11) | 0.006(13) | 0.029(10) |
| $Fe^{2+}$ | 0.001(2) | 0.030(45) | 0.188(80) | 0.007(11) | 0.103(110) | 0.099(20) |
| Mn | 0 | 0.037(52) | 0.001(1) | 0.002(1) | 0 | 0.002(0) |
| Mg | 0.021(47) | | 2.614(73) | 0.880(37) | 1.881(12) | 0.899(21) |
| Ca | 1.945(46) | 4.810(67) | 0.002(3) | 0.987(12) | 0.001(1) | |
| Ba | 0 | | 0.035(17) | | | |
| Sr | 0.031(21) | 0.058(31) | | | | |
| Na | | 0.032(28) | 0.044(22) | 0.015(5) | | |
| K | | | 0.909(15) | 0 | | |
| P | | 2.983(70) | | | | |
| La | | 0.008(9) | | | | |
| Ce | | 0.025(33) | | | | |
| F | | | 0.833(106) | | | |

[a]Number in parenthesis after the mineral is the number of EMP analyses.
[b]Number in parenthesis represents one standard deviation.
[c]below determination limits.
[d]Total iron oxide.
[e]$CO_2$ is calculated using charge balance method.
[f]$Fe^{3+}$ and $Fe^{2+}$ are calculated using charge balance method.

Supplementary Table 2: LA-ICPMS U-Th-Pb dating of apatites from Fengzhen and Huai'an carbonatites.

| Fengzhen area | | | | | | | | | | | | | |
|---|---|---|---|---|---|---|---|---|---|---|---|---|---|
| analysis # | 1 | 2 | 3 | 4 | 5 | 6 | 7 | 8 | 9 | 10 | 11 | 12 | 13 |
| $^{207}$Pb/$^{206}$Pb | 0.49302 | 0.45960 | 0.49574 | 0.48345 | 0.48072 | 0.44852 | 0.47500 | 0.50116 | 0.46484 | 0.47183 | 0.49389 | 0.46550 | 0.46776 |
| 1σ | 0.00682 | 0.00710 | 0.00688 | 0.00695 | 0.00690 | 0.00662 | 0.00698 | 0.00814 | 0.00710 | 0.00690 | 0.00910 | 0.00689 | 0.00645 |
| $^{207}$Pb/$^{235}$U | 36.65140 | 32.10383 | 37.08942 | 35.32954 | 35.04809 | 30.53121 | 34.03195 | 37.92822 | 32.76921 | 33.70228 | 36.79406 | 32.79571 | 33.27996 |
| 1σ | 0.47278 | 0.45272 | 0.48092 | 0.47048 | 0.46636 | 0.41137 | 0.46083 | 0.56626 | 0.45772 | 0.45332 | 0.61394 | 0.44506 | 0.42576 |
| $^{206}$Pb/$^{238}$U | 0.53938 | 0.50679 | 0.54281 | 0.53018 | 0.52893 | 0.49384 | 0.51976 | 0.54897 | 0.51134 | 0.51809 | 0.54034 | 0.51099 | 0.51601 |
| 1σ | 0.00997 | 0.00990 | 0.01007 | 0.00999 | 0.00996 | 0.00933 | 0.00991 | 0.01131 | 0.00995 | 0.00984 | 0.01211 | 0.00976 | 0.00944 |
| $^{208}$Pb/$^{232}$Th | 0.09765 | 0.09825 | 0.09920 | 0.09828 | 0.09800 | 0.09542 | 0.09705 | 0.10269 | 0.09467 | 0.09716 | 0.10125 | 0.09641 | 0.09626 |
| 1σ | 0.00108 | 0.00112 | 0.00110 | 0.00110 | 0.00110 | 0.00107 | 0.00109 | 0.00120 | 0.00107 | 0.00108 | 0.00124 | 0.00108 | 0.00105 |
| $^{207}$Pb correction age (Ma) | 1677 | 1685 | 1678 | 1681 | 1686 | 1678 | 1677 | 1678 | 1683 | 1682 | 1677 | 1680 | 1689 |
| 1σ | 158 | 137 | 160 | 152 | 150 | 130 | 146 | 165 | 140 | 144 | 161 | 140 | 142 |

| Fengzhen area | | | | | | | | |
|---|---|---|---|---|---|---|---|---|
| analysis # | 14 | 15 | 16 | 17 | 18 | 19 | 20 | 21 |
| $^{207}$Pb/$^{206}$Pb | 0.45523 | 0.48590 | 0.48111 | 0.45200 | 0.46089 | 0.44250 | 0.45500 | 0.47729 |
| 1σ | 0.00671 | 0.00676 | 0.00635 | 0.00707 | 0.00668 | 0.00674 | 0.00733 | 0.00645 |
| $^{207}$Pb/$^{235}$U | 31.48928 | 35.60927 | 35.10178 | 30.97223 | 32.29222 | 29.90846 | 31.42305 | 34.49001 |
| 1σ | 0.42533 | 0.46132 | 0.43496 | 0.43989 | 0.43069 | 0.41446 | 0.45901 | 0.43503 |
| $^{206}$Pb/$^{238}$U | 0.50167 | 0.53144 | 0.52906 | 0.49688 | 0.50805 | 0.49008 | 0.50075 | 0.52393 |
| 1σ | 0.00952 | 0.00985 | 0.00951 | 0.00977 | 0.00957 | 0.00945 | 0.01005 | 0.00954 |
| $^{208}$Pb/$^{232}$Th | 0.09311 | 0.09716 | 0.09747 | 0.09476 | 0.09408 | 0.09041 | 0.09192 | 0.09503 |
| 1σ | 0.00104 | 0.00106 | 0.00105 | 0.00106 | 0.00103 | 0.00101 | 0.00105 | 0.00102 |
| $^{207}$Pb correction age (Ma) | 1683 | 1677 | 1686 | 1677 | 1685 | 1684 | 1680 | 1682 |
| 1σ | 134 | 153 | 150 | 132 | 138 | 127 | 134 | 147 |

Supplementary Table 2 (Con't)

| | Huai'an area | | | | | | | | | | | | |
|---|---|---|---|---|---|---|---|---|---|---|---|---|---|
| analysis # | 1 | 2 | 3 | 4 | 5 | 6 | 7 | 8 | 9 | 10 | 11 | 12 | 13 |
| $^{207}Pb/^{206}Pb$ | 0.35271 | 0.34320 | 0.34815 | 0.35001 | 0.34776 | 0.35177 | 0.34739 | 0.32337 | 0.33931 | 0.34013 | 0.33737 | 0.33890 | 0.33802 |
| 1σ | 0.00310 | 0.00285 | 0.00279 | 0.00288 | 0.00272 | 0.00279 | 0.00298 | 0.00291 | 0.00274 | 0.00299 | 0.00324 | 0.00274 | 0.00277 |
| $^{207}Pb/^{235}U$ | 21.35568 | 20.45328 | 20.92085 | 21.01354 | 20.84741 | 21.24744 | 20.80900 | 18.73050 | 20.12593 | 20.22678 | 19.94231 | 20.03541 | 20.01515 |
| 1σ | 0.18810 | 0.17313 | 0.17278 | 0.17683 | 0.16957 | 0.17449 | 0.18017 | 0.16768 | 0.16716 | 0.17828 | 0.18729 | 0.16670 | 0.16815 |
| $^{206}Pb/^{238}U$ | 0.43824 | 0.43132 | 0.43488 | 0.43445 | 0.43377 | 0.43703 | 0.43339 | 0.41893 | 0.42897 | 0.43007 | 0.42746 | 0.42750 | 0.42816 |
| 1σ | 0.00631 | 0.00609 | 0.00609 | 0.00614 | 0.00604 | 0.00612 | 0.00621 | 0.00610 | 0.00606 | 0.00624 | 0.00639 | 0.00606 | 0.00610 |
| $^{208}Pb/^{232}Th$ | 0.09081 | 0.09072 | 0.09188 | 0.09123 | 0.09132 | 0.09135 | 0.09113 | 0.08802 | 0.09024 | 0.09071 | 0.09110 | 0.08992 | 0.08910 |
| 1σ | 0.00090 | 0.00089 | 0.00090 | 0.00089 | 0.00089 | 0.00089 | 0.00090 | 0.00088 | 0.00089 | 0.00091 | 0.00092 | 0.00089 | 0.00088 |
| $^{207}Pb$ correction age (Ma) | 1766 | 1765 | 1766 | 1759 | 1762 | 1764 | 1762 | 1767 | 1766 | 1768 | 1765 | 1761 | 1766 |
| 1σ | 82 | 77 | 79 | 80 | 79 | 81 | 79 | 70 | 76 | 77 | 76 | 76 | 75 |

| | Huai'an area | | | | |
|---|---|---|---|---|---|
| analysis # | 14 | 15 | 16 | 17 | 18 |
| $^{207}Pb/^{206}Pb$ | 0.34036 | 0.33211 | 0.33607 | 0.33634 | 0.33859 |
| 1σ | 0.00267 | 0.00288 | 0.00276 | 0.00276 | 0.00286 |
| $^{207}Pb/^{235}U$ | 20.23610 | 19.46787 | 19.81238 | 19.86678 | 20.07200 |
| 1σ | 0.16507 | 0.16993 | 0.16678 | 0.16703 | 0.17244 |
| $^{206}Pb/^{238}U$ | 0.42981 | 0.42376 | 0.42616 | 0.42697 | 0.42850 |
| 1σ | 0.00608 | 0.00617 | 0.00611 | 0.00613 | 0.00622 |
| $^{208}Pb/^{232}Th$ | 0.09165 | 0.09075 | 0.09173 | 0.08955 | 0.09164 |
| 1σ | 0.00091 | 0.00091 | 0.00092 | 0.00090 | 0.00092 |
| $^{207}Pb$ correction age (Ma) | 1766 | 1764 | 1763 | 1766 | 1766 |
| 1σ | 76 | 73 | 75 | 75 | 76 |

Supplementary Table 3: Compositions (wt%) of matrix minerals and inclusion minerals in garnets from eclogite xenolith.

| | matrix | | | | | | inclusion | | | | | | |
|---|---|---|---|---|---|---|---|---|---|---|---|---|---|
| sample | Grt(20) | Omp(9) | Ky(7) | Zo(7) | Phen(8) | Amp(7) | Omp(11) | ky(15) | Zo(10) | Rt(3) | Bar(12) | Na-amp(8) | Pg(2) |
| $SiO_2$ | 40.77(29) | 55.70(37) | 37.11(31) | 40.01(19) | 51.46(39) | 55.44(65) | 55.47(67) | 37.27(38) | 40.01(19) | 0.09(7) | 48.40(123) | 39.95(109) | 46.65(70) |
| $TiO_2$ | 0.05(3) | 0.08(1) | bdl | 0.06(2) | 0.47(3) | 0.09(2) | 0.07(1) | 0.01(1) | 0.06(2) | 97.39(43) | 0.34(10) | 0.64(7) | 0.19(4) |
| $Al_2O_3$ | 22.61(17) | 6.98(18) | 62.51(22) | 32.66(50) | 27.24(32) | 5.66(80) | 7.09(33) | 61.23(111) | 32.66(50) | 0.05(5) | 14.16(82) | 24.03(100) | 40.16(42) |
| $Cr_2O_3$ | 0.03(2) | 0.06(4) | 0.14(11) | 0.04(7) | 0.05(1) | 3.02(15) | 0.05(2) | 0.04(6) | 0.04(7) | 0.32(19) | 0.10(5) | 3.27(8) | 0.07(0) |
| $FeO^*$ | 15.97(80) | 1.52(27) | 0.08(6) | 1.07(50) | 0.62(7) | 0.06(2) | 1.51(8) | 0.59(35) | 1.07(50) | 0.16(8) | 5.31(77) | 0.05(1) | 0.54(14) |
| MnO | 0.49(8) | 0.02(1) | 0.01(1) | 0.02(2) | 0.01(1) | 0.04(3) | 0.02(2) | 0.01(1) | 0.02(2) | bdl | 0.06(1) | 0.02(1) | 0.01(2) |
| MgO | 12.22(94) | 13.24(15) | 0.01(1) | 0.08(2) | 4.01(18) | 20.61(48) | 12.78(23) | 0.15(38) | 0.08(2) | bdl | 16.35(63) | 22.25(35) | 0.35(16) |
| CaO | 7.95(45) | 19.36(27) | 0.02(2) | 24.12(14) | 0.01(1) | 11.45(28) | 19.71(27) | 0.12(23) | 24.12(14) | 0.02(2) | 9.51(35) | 0.12(3) | 1.04(23) |
| $Na_2O$ | 0.02(1) | 3.21(10) | 0.02(1) | 0.01(1) | 0.52(6) | 1.47(11) | 3.44(14) | 0.01(0) | 0.01(1) | bdl | 2.58(29) | 5.15(37) | 6.79(40) |
| $K_2O$ | 0.02(0) | 0.01(1) | 0.01(2) | 0.01(1) | 10.04(17) | 0.23(4) | 0.01(2) | 0.05(14) | 0.01(1) | bdl | 0.10(5) | 0.31(3) | 0.33(21) |
| F | | | | | 0.10(2) | | | | | | | | |
| Cl | | | | | 0.01(1) | | | | | | | | |
| O=F,Cl | | | | | -0.04(1) | | | | | | | | |
| Total | 100.14(18) | 100.19(35) | 99.91(25) | 98.08(30) | 94.51(36) | 98.06(41) | 100.15(72) | 99.81(20) | 98.08(30) | 99.39(27) | 96.91(41) | 95.77(36) | 96.12(106) |
| *Cations* | *6O* | *6O* | *5O* | | | | *6O* | *5O* | | *2O* | | | *6O* |
| Si | 3.019(5) | 1.989(9) | 1.001(7) | 3.027(7) | 3.425(13) | 7.547(69) | 1.980(14) | 1.000(7) | 3.027(7) | 0.001(1) | 6.673(125) | 4.960(109) | 2.957(6) |
| Ti | 0.003(2) | 0.002(0) | 0 | 0.003(1) | 0.023(1) | 0.009(2) | 0.002(0) | 0 | 0.003(1) | 0.985(5) | 0.036(11) | 0.060(7) | 0.009(2) |
| Al | 1.973(9) | 0.293(8) | 1.957(69) | 2.913(35) | 2.137(26) | 0.907(132) | 0.298(13) | 1.951(17) | 2.913(35) | 0.001(1) | 2.300(144) | 3.515(163) | 3.001(8) |
| Cr | 0.002(1) | 0.002(1) | 0.003(3) | 0.003(4) | 0.003(1) | 0.007(3) | 0.001(1) | 0.001(1) | 0.003(4) | 0.004(2) | 0.011(6) | 0.005(1) | 0.003(0) |
| $Fe^{3+}$ | 0 | 0 | 0 | 0.060(37) | 0 | 0.203(46) | 0 | 0 | 0.060(37) | | 0.613(93) | 0.339(7) | 0 |
| $Fe^{2+}$ | 0.989(55) | 0.045(8) | 0.008(14) | 0.008(15) | 0.034(4) | 0.140(47) | 0.045(2) | 0.010(5) | 0.008(15) | 0.002(1) | 0 | 0 | 0.029(7) |
| Mn | 0.031(5) | 0.001(1) | 0 | 0.001(1) | 0 | 0.004(4) | 0.001(1) | 0 | 0.001(1) | | 0.007(1) | 0.002(1) | 0.001(1) |
| Mg | 1.348(95) | 0.704(7) | 0 | 0.009(2) | 0.398(18) | 4.182(84) | 0.680(15) | 0.001(1) | 0.009(2) | 0 | 3.360(108) | 4.119(42) | 0.033(15) |
| Ca | 0.631(39) | 0.741(13) | 0.004(10) | 1.955(11) | 0.001(1) | 1.670(35) | 0.754(13) | 0.002(2) | 1.955(11) | 0.001(1) | 1.406(50) | 0.015(4) | 0.071(15) |
| Na | 0.003(1) | 0.222(6) | 0.001(1) | 0.001(1) | 0.067(8) | 0.389(30) | 0.238(9) | 0 | 0.001(1) | | 0.691(79) | 1.240(94) | 0.835(61) |
| K | 0.002(1) | 0.001(1) | 0 | 0.002(1) | 0.853(16) | 0.041(7) | 0 | 0.001(1) | 0.002(1) | | 0.017(9) | 0.048(4) | 0.027(17) |

Grt, garnet; Omp, omphacite; Ky, kyanite; Zo, zoisite; Phen, phengite; Amp, amphibole; Rt, rutile; Bar, barroisite; Pg, paragonite.

Supplementary Table 4: U-Th-Pb ages of monazite in garnets of eclogite xenolith.

| sample | U(ppm) | error | Pb(ppm) | error | Th*(ppm) | error | Age(Ma) | 2σ |
|---|---|---|---|---|---|---|---|---|
| Primary crystal | | | | | | | | |
| M-1 | 2236 | 390 | 3739 | 240 | 42179 | 720 | 1856 | 79 |
| M-2 | 2348 | 180 | 4343 | 110 | 50255 | 430 | 1818 | 31 |
| M-3 | 3925 | 190 | 4672 | 120 | 53865 | 420 | 1803 | 31 |
| M-4 | 4305 | 190 | 4947 | 120 | 55891 | 420 | 1833 | 30 |
| M-5 | 5964 | 200 | 5542 | 120 | 60420 | 420 | 1874 | 27 |
| M-6 | 5085 | 200 | 5251 | 120 | 58339 | 420 | 1852 | 28 |
| In calcite veinlet | | | | | | | | |
| M-7 | 835 | 380 | 5104 | 260 | 62242 | 940 | 1758 | 60 |
| M-8 | 775 | 170 | 4757 | 120 | 58072 | 510 | 1756 | 30 |
| M-9 | 784 | 170 | 4608 | 120 | 56611 | 500 | 1746 | 30 |
| M-10 | 986 | 170 | 4876 | 120 | 59491 | 510 | 1755 | 29 |
| M-11 | 869 | 170 | 5302 | 120 | 64409 | 550 | 1765 | 27 |
| M-12 | 867 | 170 | 4945 | 120 | 59813 | 520 | 1771 | 29 |
| M-13 | 1242 | 180 | 6601 | 130 | 79564 | 630 | 1776 | 23 |
| M-14 | 964 | 180 | 8157 | 140 | 99012 | 760 | 1769 | 20 |
| M-15 | 864 | 180 | 8237 | 140 | 99533 | 760 | 1778 | 20 |
| M-16 | 1618 | 180 | 7033 | 130 | 85789 | 660 | 1753 | 22 |
| M-17 | 3024 | 180 | 3150 | 110 | 36913 | 330 | 1769 | 41 |
| M-18 | 3747 | 190 | 4598 | 120 | 53949 | 420 | 1777 | 31 |

Th*, apparent Th, knowing the age of each point, the U content, converted into the equivalent Th content that would have produced the same amount of Pb, was added to the measured Th to give the amount of apparent Th.

Supplementary Table 5: Mineral and modeling compositions of eclogite xenolith.

| Mineral | Volume(%) | Mass(%) | Modeling composition | |
|---|---|---|---|---|
| omphacite | 36.50 | 34.59 | $SiO_2$ | 49.21 |
| garnet | 35.90 | 41.54 | $TiO_2$ | 0.14 |
| kyanite | 8.76 | 9.0 | $Al_2O_3$ | 20.09 |
| quartz | 8.60 | 6.50 | FeO | 6.93 |
| zoisite | 7.20 | 5.70 | MgO | 10.16 |
| phengite | 1.52 | 1.21 | CaO | 11.86 |
| amphibole | 0.87 | 0.79 | MnO | 0.20 |
| biotite | 0.39 | 0.35 | $Na_2O$ | 0.99 |
| rutile | 0.10 | 0.12 | $K_2O$ | 0.17 |
| rest | 0.16 | 0.20 | $H_2O$ | 0.22 |
| | | | O | 0.02 |
| total | 100.00 | 100.00 | | 99.99 |

Supplementary Table 6: trace element compositions (ppm) of garnet, omphacite, kyanite, zoisite, phengite in eclogite xenolith, and of calculated whole-rock

| Sample | Garnet n=9 | Omphacite n=8 | Kyanite n=5 | Zoisite n=10 | Phengite n=3 | Reconstructed bulk rock |
|---|---|---|---|---|---|---|
| Rb | 0.02(3) | 0.06(13) | bdl | 0.01(2) | 195(10) | 2.38 |
| Sr | 0.04(4) | 55.0(64) | 0.03(5) | 3757(153) | 105(22) | 234 |
| Y | 24.1(71) | 0.25(6) | bdl | 12.0(20) | 0.13(2) | 10.8 |
| Zr | 2.57(103) | 2.73(15) | 0.01(2) | 1.07(36) | 0.31(3) | 2.08 |
| Nb | bdl | bdl | bdl | bdl | 0.11(3) | bdl |
| Ba | bdl | 0.02(5) | bdl | 5.62(48) | 2514(75) | 30.7 |
| La | bdl | bdl | bdl | 19.0(46) | 0.01(2) | 1.09 |
| Ce | 0.01(2) | bdl | 0.02(2) | 42.7(101) | 0.01(2) | 2.44 |
| Pr | bdl | bdl | bdl | 5.21(118) | bdl | 0.30 |
| Nd | 0.02(2) | bdl | 0.02(5) | 22.6(46) | bdl | 1.30 |
| Sm | 0.02(4) | 0.01(2) | 0.03(7) | 6.47(112) | bdl | 0.38 |
| Eu | 0.06(2) | 0.02(1) | bdl | 7.26(98) | bdl | 0.44 |
| Gd | 0.21(9) | bdl | bdl | 7.17(109) | bdl | 0.50 |
| Tb | 0.14(6) | 0.01(1) | bdl | 1.01(15) | bdl | 0.12 |
| Dy | 2.68(113) | 0.05(3) | bdl | 5.23(79) | bdl | 1.43 |
| Ho | 0.93(30) | 0.01(1) | bdl | 0.55(8) | bdl | 0.42 |
| Er | 3.06(66) | 0.01(2) | bdl | 0.57(13) | bdl | 1.31 |
| Tm | 0.41(10) | 0.01(1) | bdl | 0.02(2) | bdl | 0.17 |
| Yb | 2.56(107) | 0.01(2) | bdl | 0.05(9) | bdl | 1.07 |
| Lu | 0.35(19) | bdl | bdl | bdl | bdl | 0.15 |
| Hf | 0.02(3) | 0.20(3) | bdl | 0.02(6) | 0.03(5) | 0.08 |
| Ta | bdl | 0.01(1) | bdl | bdl | 0.04(4) | bdl |
| Pb | bdl | bdl | bdl | 10.7(5) | 0.42(15) | 0.61 |
| Th | bdl | bdl | 0.02(3) | 5.75(270) | 0.01(2) | 0.33 |
| U | bdl | bdl | 0.01(2) | 1.43(55) | bdl | 0.08 |

Supplementary Table 7: Chemical compositions of rutiles (ppm except for Ti, O and Total with wt.%) in the matrix of eclogite.

| | Rt-1 | | Rt-2 | | Rt-3 | | Rt-4 | | Rt-5 | Rt-6 | Rt-7 | Rt-8 | Rt-9 | Rt-10 | Rt-11 |
|---|---|---|---|---|---|---|---|---|---|---|---|---|---|---|---|
| | rim | core | rim | core | rim | core | rim | core | | | | | | | |
| Si | 23(19) | 75(20) | 71(20) | 116(20) | 61(20) | 23(19) | 129(20) | 66(20) | 31(20) | 43(20) | 60(20) | 128(20) | 95(20) | 476(22) | 134 (20) |
| Fe | 995(35) | 1852(40) | 2903(47) | 3406(50) | 924(35) | 750(33) | 2586(45) | 1994(41) | 649(33) | 1789(40) | 1817(40) | 789(33) | 2491(44) | 1534(38) | 2195(43) |
| Cr | 1487(37) | 1474(37) | 1275(35) | 1245(35) | 1311(36) | 1310(36) | 1665(38) | 1630(38) | 1292(36) | 1978(40) | 2143(41) | 1509(37) | 1980(40) | 1695(39 | 1654(39) |
| Zr | 147(30) | 141(30) | 152(31) | 148(31) | 160(31) | 157(30) | 148(31) | 143(31) | 146(31) | 174(30) | 151(31) | 145(30) | 145(30) | 168(31) | 148(31) |
| V | 2018(23) | 2037(23) | 1966(23) | 1967(23) | 1667(20) | 1680(20) | 1951(23) | 1927(22) | 1723(20) | 1834(21) | 1817(21) | 1836(21) | 2005(23) | 1629(19) | 1655(20) |
| Nb | 89(47) | 109(47) | 117(47) | 98(47) | 81(47) | 98(47) | 120(47) | 142(47) | 73(47) | 106(47) | 94(47) | 131(46) | 83(47) | 111(48) | 88 (48) |
| Ta | bdl | bdl | bdl | bdl | bdl | bdl | bdl | bdl | bdl | bdl | bdl | bdl | bdl | bdl | bdl |
| Ti | 59.10(20) | 59.34(20) | 59.37(20) | 58.84(20) | 58.96(20) | 59.31(20) | 59.25(20) | 59.44(20) | 59.40(20) | 58.96(20) | 59.02(20) | 59.29(20) | 59.24(20) | 59.38(20) | 59.09(20) |
| O | 39.68 | 39.87 | 39.91 | 39.58 | 39.57 | 39.79 | 39.84 | 39.95 | 39.85 | 39.63 | 39.68 | 39.81 | 39.85 | 39.93 | 39.71 |
| Total | 99.26 | 99.78 | 99.93 | 99.11 | 98.95 | 99.50 | 99.75 | 99.99 | 99.64 | 99.19 | 99.31 | 99.56 | 99.78 | 99.87 | 99.39 |

| | Rt-12 | Rt-13 | Rt-14 | Rt-15 | Rt-16 | Rt-17 | Rt-18 | Rt-19 | Rt-20 | Rt-21 | Rt-22 | Rt-23 | Rt-24 | Rt-25 | Rt-26 |
|---|---|---|---|---|---|---|---|---|---|---|---|---|---|---|---|
| Si | 41(20) | 55(20) | 148(20) | 57(20) | 44(20) | 60(20) | 44(20) | 207(21) | 131(20) | 99(19) | 52(18) | 57(18) | 51(18) | 63(18) | 62(18) |
| Fe | 766(34) | 715(34) | 3535(34) | 603(33) | 631(33) | 1160(37) | 789(34) | 4358(37) | 778(34) | 976(33) | 905(32) | 832(32) | 795(32) | 751(31) | 817(32) |
| Cr | 1479(37) | 1735(39) | 1430(37) | 1707(39) | 2291(42) | 2695(44) | 1449(37) | 1601(38) | 2578(44) | 1512(35) | 1567(35) | 1554(35) | 1541(35) | 1545(35) | 1516(35) |
| Zr | 146(31) | 162(31) | 145(30) | 162(31) | 144(31) | 148(31) | 144(31) | 144(31) | 146(31) | 141(28) | 152(29) | 143(28) | 153(28) | 150(28) | 161(29) |
| V | 2024(23) | 2047(23) | 1999(23) | 2123(25) | 2216(25) | 2047(24) | 2006(23) | 2171(25) | 1993(23) | 1736(19) | 1720(19) | 1772(19) | 1749(19) | 1721(19) | 1720(19) |
| Nb | 122(47) | 96(47) | 96(47) | 92(47) | 109(47) | 124(48) | 89(48) | 103(48) | 87(48) | 82(43) | 83(43) | 88(43) | 106(43) | 94(44) | 88(43) |
| Ta | bdl | bdl | bdl | bdl | bdl | bdl | bdl | bdl | bdl | bdl | bdl | bdl | bdl | bdl | bdl |
| Ti | 59.51(20) | 59.62(20) | 58.74(20) | 59.14(20) | 59.14(20) | 59.09(20) | 59.22(20) | 58.73(20) | 58.93(20) | 59.53(19) | 59.79(19) | 59.97(19) | 60.06(19) | 59.90(19) | 59.99(19) |
| O | 39.95 | 40.04 | 39.52 | 39.72 | 39.75 | 39.75 | 39.76 | 39.57 | 39.63 | 39.96 | 40.13 | 40.25 | 40.31 | 40.21 | 40.27 |
| Total | 99.93 | 100.14 | 98.99 | 99.34 | 99.44 | 99.47 | 99.44 | 99.16 | 99.13 | 99.95 | 100.37 | 100.66 | 100.81 | 100.55 | 100.70 |

Supplementary Table 8: Peak P-T calculation of eclogite xenolith.

| | Zr[a] | T($^{o}$C)[b] | T($^{o}$C)[c] | T($^{o}$C)[d] | Waters P(kbar)[e] | |
|---|---|---|---|---|---|---|
| | ppm in max, min | | P=25 kbar | P=25 kbar | T=670(23) $^{o}$C | T=650(7) $^{o}$C |
| Zr-in-rutile thermometry | 174(30) | 670(23) | 669(14) | | | |
| | 150(8) | 650(7) | 658(4) | | | |
| Grt-Cpx thermometry | | | | 670 | | |
| Grt-Cpx-Phen barometer | | | | | 24.6(0.4) | 24.9(0.4) |

[a]the maximum and minimum Zr contents of rutile were analyzed from the eclogitic matrix in Supplementary Table 7.
[b]Zr-in-rutile thermometer[2] is pressure independence.
[c]Zr-in-rutile thermometer[3] was calculated at pressure dependence.
[d]due to the end member of grossular in the eclogitic garnet is less than 0.35, Carswell et al.[4] suggested that the garnet-clinopyroxene (Grt-Cpx) thermometer preferentially use the formula in Powell[5]. The garnet rim and average omphacite in the matrix were chosen as peak mineral assemblage to give a mean temperature.
[e]because of low jadeite content (<0.5) of omphacite, Chen et al.[6] suggested the garnet-clinopyroxene-phengite (Grt-Cpx-Phen) barometer preferentially choose the formula in Ref. 7. The garnet rim and average omphacite in the matrix and phengite with high Si (3.44 afu) were chosen as peak mineral assemblage. The temperature values were chosen from the Zr-in-rutile thermometer.

Supplementary Table 9: C, O isotopes of carbonatites from Fengzhen and Huai'an.

| | Fengzhen area | | | | | | | | | | Huai'an area | | | | |
|---|---|---|---|---|---|---|---|---|---|---|---|---|---|---|---|
| analysis # | 1 | 2 | 3 | 4 | 5 | 6 | 7 | 8 | 9 | 10 | 1 | 2 | 3 | 4 | 5 |
| $\delta^{13}C_{PDB}$ (‰) | -4.30 | -2.86 | -5.21 | -1.59 | -4.02 | -5.12 | -5.71 | -3.23 | -3.24 | -3.24 | -3.27 | -3.06 | -3.23 | -5.99 | -3.16 |
| $\delta^{18}O_{SMOW}$ (‰) | 11.6 | 11.92 | 12.93 | 14.38 | 13.02 | 12.32 | 12.37 | 15.82 | 15.22 | 15.82 | 9.47 | 9.56 | 9.38 | 14.55 | 9.43 |

Supplementary Table 10: Sr-Nd isotopes of carbonatites and apatites from Fengzhen and Huai'an.

| carbonatite | Fengzhen area | | | | | | Huai’an area | | |
|---|---|---|---|---|---|---|---|---|---|
| analysis # | 1 | 2 | 3 | 4 | 5 | 6 | 1 | 2 | 3 |
| Rb (ppm) | 1.11 | 7.33 | 1.74 | 0.353 | 64.1 | 0.893 | 0.529 | 0.566 | 0.492 |
| Sr (ppm) | 16744 | 7159 | 4515 | 2753 | 6308 | 17042 | 10427 | 10750 | 2209 |
| $^{87}Rb/^{86}Sr$ | 0.0002 | 0.0030 | 0.0011 | 0.0004 | 0.0290 | 0.0001 | 0.0001 | 0.0002 | 0.0006 |
| $^{87}Sr/^{86}Sr$ | 0.702802 | 0.703092 | 0.702952 | 0.703659 | 0.703659 | 0.702804 | 0.703191 | 0.703218 | 0.703606 |
| ±2s | 10 | 7 | 13 | 6 | 6 | 14 | 8 | 11 | 11 |
| $(^{87}Sr/^{86}Sr)_i^a$ | 0.70280 | 0.70302 | 0.70292 | 0.70365 | 0.70295 | 0.70280 | 0.70319 | 0.70321 | 0.70359 |
| Sm (ppm) | 129 | 28 | 32.1 | 130 | 60.1 | 101 | 34.9 | 33.8 | 14.3 |
| Nd (ppm) | 1274 | 252 | 215 | 1335 | 535 | 1039 | 271 | 261 | 139 |
| $^{147}Sm/^{144}Nd$ | 0.0612 | 0.0672 | 0.0903 | 0.0589 | 0.0679 | 0.0588 | 0.0778 | 0.0783 | 0.0622 |
| $^{143}Nd/^{144}Nd$ | 0.510808 | 0.510847 | 0.511139 | 0.510757 | 0.510874 | 0.510752 | 0.510993 | 0.511018 | 0.510736 |
| ±2s | 13 | 11 | 18 | 15 | 16 | 14 | 17 | 15 | 14 |
| $(^{143}Nd/^{144}Nd)_i^a$ | 0.510124 | 0.510096 | 0.510129 | 0.510098 | 0.510115 | 0.510095 | 0.510123 | 0.510142 | 0.510041 |
| $\varepsilon_{Nd}(t)^b$ | -6.2 | -6.7 | -6.1 | -6.7 | -6.3 | -6.7 | -6.2 | -5.8 | -7.8 |
| T (model age, Ga) | 2.33 | 2.38 | 2.47 | 2.35 | 2.37 | 2.35 | 2.41 | 2.39 | 2.42 |

Supplementary Table 10 (Con't)

| apatite | Fengzhen area | | | | | | | | | | | | | | |
|---|---|---|---|---|---|---|---|---|---|---|---|---|---|---|---|
| analysis # | 1 | 2 | 3 | 4 | 5 | 6 | 7 | 8 | 9 | 10 | 11 | 12 | 13 | 14 | 15 |
| $^{87}Rb/^{86}Sr$ | 1E-05 | 8E-06 | 9E-07 | 2E-06 | 1E-06 | 3E-06 | 4E-06 | 1E-07 | 5E-06 | 5E-06 | 6E-06 | 4E-06 | 3E-06 | 1E-06 | 1E-05 |
| ±2s | 0.8 | 8 | 84 | 8 | 8 | 8 | 8 | 74 | 7 | 8 | 7 | 7 | 7 | 7 | 0.8 |
| $^{87}Sr/^{86}Sr$ | 0.70295 | 0.70294 | 0.70291 | 0.70301 | 0.70303 | 0.70301 | 0.70296 | 0.70300 | 0.70303 | 0.70292 | 0.70293 | 0.70298 | 0.70299 | 0.70298 | 0.70301 |
| ±2s | 6 | 10 | 5 | 7 | 6 | 6 | 6 | 6 | 6 | 5 | 5 | 5 | 5 | 5 | 5 |
| $(^{87}Sr/^{86}Sr)_i$ [a] | 0.70295 | 0.70294 | 0.70291 | 0.70301 | 0.70303 | 0.70301 | 0.70296 | 0.70300 | 0.70303 | 0.70292 | 0.70293 | 0.70298 | 0.70299 | 0.70298 | 0.70301 |
| $^{147}Sm/^{144}Nd$ | 0.07994 | 0.08018 | 0.07989 | 0.07958 | 0.07949 | 0.07936 | 0.07927 | 0.07922 | 0.08001 | 0.07990 | 0.07982 | 0.07977 | 0.07955 | 0.07958 | 0.08029 |
| ±2s | 3 | 3 | 3 | 3 | 3 | 3 | 3 | 3 | 2 | 3 | 3 | 3 | 3 | 3 | 4 |
| $^{143}Nd/^{144}Nd$ | 0.511033 | 0.511004 | 0.511013 | 0.511013 | 0.511041 | 0.510996 | 0.511016 | 0.511015 | 0.511038 | 0.510957 | 0.510989 | 0.511042 | 0.510973 | 0.510986 | 0.511029 |
| ±2s | 30 | 30 | 30 | 30 | 30 | 30 | 30 | 30 | 20 | 30 | 30 | 30 | 30 | 30 | 30 |
| $(^{143}Nd/^{144}Nd)_i$ [a] | 0.510140 | 0.510107 | 0.510120 | 0.510123 | 0.510152 | 0.510109 | 0.510130 | 0.510130 | 0.510143 | 0.510064 | 0.510097 | 0.510150 | 0.510083 | 0.510096 | 0.510131 |
| $\varepsilon_{Nd}(t)$ [b] | -5.86 | -6.49 | -6.26 | -6.18 | -5.61 | -6.47 | -6.05 | -6.06 | -5.79 | -7.35 | -6.70 | -5.65 | -6.97 | -6.71 | -6.03 |
| T (Ga) | 2.40 | 2.44 | 2.42 | 2.42 | 2.38 | 2.43 | 2.41 | 2.41 | 2.40 | 2.49 | 2.45 | 2.39 | 2.46 | 2.45 | 2.41 |

| apatite | Huai'an area | | | | | | | | | | | | | | | |
|---|---|---|---|---|---|---|---|---|---|---|---|---|---|---|---|---|
| analysis # | 1 | 2 | 3 | 4 | 5 | 6 | 7 | 8 | 9 | 10 | 11 | 12 | 13 | 14 | 15 | 16 |
| $^{87}Rb/^{86}Sr$ | 2E-05 | 2E-05 | 3E-05 | 1E-05 | 2E-05 | 3E-05 | 2E-05 | 1E-05 | 2E-05 | 2E-05 | 1E-05 | 2E-05 | 2E-05 | 2E-05 | 2E-05 | 1E-05 |
| ±2s | 0.6 | 0.8 | 0.7 | 0.8 | 0.8 | 0.7 | 0.7 | 0.7 | 0.7 | 0.6 | 0.7 | 0.7 | 0.9 | 0.9 | 0.9 | 0.6 |
| $^{87}Sr/^{86}Sr$ | 0.70277 | 0.70270 | 0.70282 | 0.70281 | 0.70280 | 0.70270 | 0.70275 | 0.70274 | 0.70282 | 0.70274 | 0.70278 | 0.70276 | 0.70281 | 0.70275 | 0.70282 | 0.70277 |
| ±2s | 7 | 6 | 6 | 7 | 6 | 6 | 6 | 6 | 5 | 7 | 5 | 6 | 6 | 7 | 6 | 6 |
| $(^{87}Sr/^{86}Sr)_i$ [a] | 0.70276 | 0.70270 | 0.70282 | 0.70281 | 0.70280 | 0.70270 | 0.70275 | 0.70274 | 0.70282 | 0.70274 | 0.70278 | 0.70276 | 0.70281 | 0.70275 | 0.70282 | 0.70277 |
| $^{147}Sm/^{144}Nd$ | 0.07523 | 0.07521 | 0.07471 | 0.07711 | 0.07752 | 0.07679 | 0.07455 | 0.07624 | 0.07615 | 0.07621 | 0.07714 | 0.07648 | 0.07680 | 0.07630 | 0.07628 | 0.07629 |
| ±2s | 4 | 4 | 4 | 3 | 3 | 4 | 3 | 3 | 4 | 4 | 4 | 4 | 5 | 4 | 3 | 4 |
| $^{143}Nd/^{144}Nd$ | 0.510959 | 0.510976 | 0.510945 | 0.510993 | 0.511006 | 0.510963 | 0.510955 | 0.510969 | 0.510963 | 0.510989 | 0.510976 | 0.510991 | 0.510935 | 0.510952 | 0.510983 | 0.510960 |
| ±2s | 30 | 30 | 40 | 30 | 30 | 30 | 30 | 30 | 30 | 30 | 30 | 30 | 40 | 30 | 30 | 30 |
| $(^{143}Nd/^{144}Nd)_i$ [a] | 0.510118 | 0.510135 | 0.510109 | 0.510131 | 0.510139 | 0.510105 | 0.510121 | 0.510116 | 0.510112 | 0.510137 | 0.510114 | 0.510136 | 0.510076 | 0.510099 | 0.510130 | 0.510107 |
| $\varepsilon_{Nd}(t)$ [b] | -6.28 | -5.95 | -6.45 | -6.03 | -5.87 | -6.55 | -6.22 | -6.32 | -6.41 | -5.91 | -6.37 | -5.94 | -7.10 | -6.67 | -6.04 | -6.50 |
| T (Ga) | 2.40 | 2.38 | 2.41 | 2.40 | 2.39 | 2.42 | 2.39 | 2.41 | 2.41 | 2.38 | 2.41 | 2.39 | 2.45 | 2.43 | 2.39 | 2.42 |

[a] Initial Sr, Nd isotopic ratios of the carbonatites are calculated assuming an age of 1800 Ma.

[b] $\varepsilon_{Nd}(t)$ values are calculated based on present-day $(^{147}Sm/^{143}Nd)CHUR = 0.1967$ and $(^{143}Nd/^{144}Nd)CHUR = 0.512638$.

Supplementary Table 11: components of end-members used in carbonatite source model.

| Composition | Old altered ocean crust | Old marine sediment |
|---|---|---|
| Rb (ppm) | 1.3 | 65 |
| Sr (ppm) | 113 | 300 |
| Sm (ppm) | 3.75 | 18 |
| Nd (ppm) | 11.2 | 85 |
| $^{87}Rb/^{86}Sr$ | 0.032 | 0.61 |
| $^{147}Sm/^{144}Nd$ | 0.19 | 0.13 |
| *Isotopes at 2.5 Ga* | | |
| $^{87}Sr/^{86}Sr$ | 0.70060 | 0.70463 |
| $^{143}Nd/^{144}Nd$ | 0.509589 | 0.509161 |
| *Isotopes at 1.8 Ga* | | |
| $^{87}Sr/^{86}Sr$ | 0.70092 | 0.71072 |
| $^{143}Nd/^{144}Nd$ | 0.510461 | 0.509757 |

The components of altered ocean crust and marine sediment are from Ref. 8. The isotopic compositions of ocean crust and marine sediment at 2.5 Ga were calculated using a single-stage evolution model with $^{87}Rb/^{86}Sr$ and $^{147}Sm/^{144}Nd$ at 0.053, 0.186 and 0.214, 0.183, respectively.

Supplementary Table 12: High-pressure metamorphic rocks in Paleoproterozoic (1.7 -2.1 Ga) orogens worldwide.

| Orogenic belt | Metamorphic rock | Area |
|---|---|---|
| Trans-Hudson | eclogite | |
| Taltson-Thelon | eclogite | |
| Penokean | granulite | |
| Cape Smith-New Quebec | granulite | |
| Torngat | granulite | |
| Foxe | granulite | |
| Nagssugtoqidian | granulite | |
| Makkovikian-Ketilidian | granulite | Canada |
| Transamazonian | granulite | Brazil |
| Eburnian | granulite | Liberia |
| Eburnian | eclogite | Cameroon |
| Ubendian | eclogite | Tanzania |
| Limpopo | granulite | South Africa |
| Capricorn | granulite | Australia |
| Moyar | granulite | India |
| Trans-North China | eclogite | China |
| Central Aldan | granulite | Russia |
| Lapland-Kola | eclogite | |
| Transantartic | granulite | Finland |

Data are cited from Refs. 9-14 and this study.

## Supplementary References


1. Jacob, D. E. Nature and origin of eclogite xenoliths from kimberlites. *Lithos* **77**, 295-316 (2004).
2. Zack, T., Moraes, R. & Kronz, A. Temperature dependence of Zr in rutile: empirical calibration of a rutile thermometer. *Contrib. Mineral. Petrol*. **148**, 471-488 (2004).
3. Tomkins, H. S., Powell, R. & Ellis, D. J. The pressure dependence of the zirconium-in-rutile thermometer. *J. Metamorph. Geol.* **25**, 703-713 (2007).
4. Carswell, D. A., O'Brien, P. J. Wilson, R. N. & Zhai, M. Thermobarometry of phengite-bearing eclogites in the Dabie Mountains of central China. *J. Metamorph. Geol.***15**, 239-252 (1997).
5. Powell, R., 1985. Regression diagnostics and robust regression in geothermometer/geobarometer calibration: the garnet-clinopyroxene geothermometer revisited. *J. Metamorph. Geol.* **3**, 231-243 (1985).
6. Chen, Y., Ye, K. & Wu, C. M. Reviews on applying common-used geothermobarometers for eclogites. *Acta Petrol. Sinica* **21**, 1067-1080 (2005).
7. Waters, D. J. & Martin, H. N. Geobarometry of phengite-bearing eclogites. *Terra Abstracts* **5**, 410-411 (1993).
8. Rehkämper, M. & Hofmann, A. W. Recycled ocean crust and sediment in Indian Ocean MORB. *Earth Planet. Sci. Lett*. **147**, 93-106 (1997).
9. Weller, O. M. & St-Onge, M. R. Record of modern-style plate tectonics in the Paleoproterozoic Trans-Hudson orogen. *Nat. Geosci.* **10**, 305-311 (2017).

10. Zhao, G. C., Cawood, P. A., Wilde, S. A. & Sun, M. Review of global 2.1-1.8 Ga orogens: implications for a pre-Rodinia supercontinent. *Earth Sci. Rev.***59**, 125-162 (2002).
11. Heaman, L. M., Creaser, R. A. & Cookenboo, H. O. Extreme enrichment of high field strength elements in Jericho eclogite xenoliths: A cryptic record of Paleoproterozoic subduction, partial melting, and metasomatism beneath the Slave craton, Canada. *Geology* **30**, 507-510 (2002).
12. Baldwin, J. A., Bowring, S. A., Williams, M. L. & Williams, I. S. Eclogites of the Snowbird tectonic zone: petrological and U-Pb geochronological evidence for Paleoproterozoic high-pressure metamorphism in the western Canadian Shield. *Contrib. Mineral. Petrol*. **147**, 528-548 (2004).
13. Boniface, N., Schenk, V. & Appel, P. Paleoproterozoic eclogites of MORB-type chemistry and three Proterozoic orogenic cycles in the Ubendian Belt (Tanzania): Evidence from monazite and zircon geochronology, and geochemistry. *Precambr. Res.* **192-195**, 16-33 (2012).
14. Liu, F. L. *et al*. The metamorphic evolution of Paleoproterozoic eclogites in Kuru-Vaara, northern Belomorian Province, Russia: Constraints from P-T pseudosections and zircon dating. *Precambr. Res*. **289**, 31-47 (2017).
